# Supplementary material for: The interactome of intact mitochondria by cross-linking mass spectrometry provides evidence for coexisting respiratory supercomplexes
Source: Mol Cell Proteomics. 2017 Dec 8;17(2):216–32. doi: 10.1074/mcp.RA117.000470 (PMC5795388; doi:10.1074/mcp.RA117.000470)
Supplement: Supplemental Data [file supp_RA117.000470_133922_0_supp_23978_fzffwf.zip › spectra_annotation/mito_spectra_annotation/123-1-7-1-8-1.pdf]

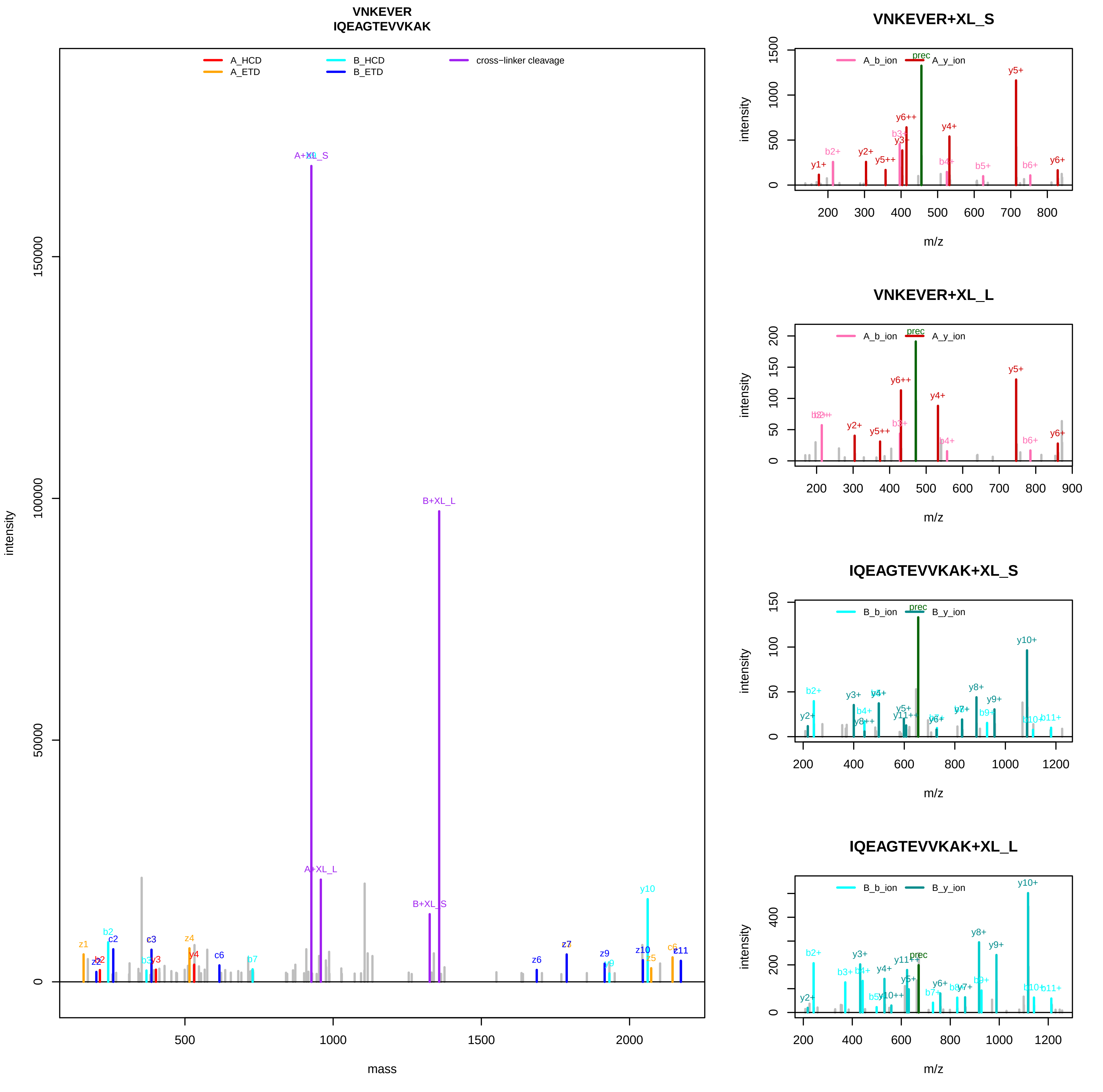

VNKEVER
IQEAGTEVVKAK
A_HCD
A_ETD
B_HCD
B_ETD
cross−linker cleavage
A+XL_S
B+XL_L
A+XL_L
B+XL_S
intensity
mass
0
50000
100000
150000
500
1000
1500
2000
z1
b2
c2
z2
c3
b3
y3
z4
y4
c6
b7
z6
z7
z9
y9
z10
y10
z5
c6
z11
VNKEVER+XL_S
VNKEVER+XL_L
IQEAGTEVVKAK+XL_S
IQEAGTEVVKAK+XL_L
A_b_ion
A_y_ion
B_b_ion
B_y_ion
prec
y1+
b2+
y2+
y5++
b3+
y3+
y6++
y4+
b4+
b5+
y5+
b6+
y6+
y3+
y4+
b4+
y8++
y5+
y11++
y6+
y7+
y8+
y9+
b9+
y10+
b10+
b11+
y2+
b2+
b3+
b5+
y10++
b7+
b8+
m/z
